# Molecular docking and dynamics simulation analysis of nucleoprotein from the Crimea-Congo hemorrhagic fever virus strain Baghdad-12 with FDA approved drugs

**DOI:** 10.6026/97320630018442

**Published:** 2022-05-31

**Authors:** Ahmed Fadhil Hashim, Hasanain Abdulhameed Odhar, Salam Waheed Ahjel

**Affiliations:** 1 Department of pharmacy, Al-Zahrawi University College, Karbala, Iraq

**Keywords:** Crimea-Congo hemorrhagic fever virus strain Baghdad-12, nucleoprotein, repurpose, docking, dynamics simulation

## Abstract

Crimea-Congo hemorrhagic fever virus is considered a potential public health threat due to the high case fatality ratio of the disease hemorrhagic phase and absence of approved vaccines or antiviral agents. Therefore, it is of interest to screen FDA approved drugs against the nucleoprotein crystal of Crimea-Congo hemorrhagic fever virus strain Baghdad-12 by using molecular docking and dynamics simulation. Hence, we report that the beta receptor blocker Nebivolol and the antihistamine Loratadine may bind to RNA binding region on nucleoprotein for further consideration in drug design and development.

## Background:

Crimea-Congo hemorrhagic fever virus (CCHFV) is a single-stranded and negative sense RNA pathogen that is capable of causing a serious hemorrhagic fever in some infected individual. This severe hemorrhagic fever is known as Crimea-Congo hemorrhagic fever (CCHF) disease and the reported case fatality ratio of this disease is 10%-40% [1-3]. Although infections with CCHFV have been documented in more than 30 countries, most of these cases are believed to be mild or sub clinical [1,4]. However, CCHFV infection in some patients can be fatal as the disease can progress into a severe form of hemorrhagic fever. This variation in clinical severity of CCHF disease may be attributed partially to the polymorphism in innate immune system components like Toll-like receptors (TLRs) [5]. Furthermore, the exploration of CCHF pathogenesis is considered challenging due to the lack of a convenient animal model [6]. CCHFV is usually considered a tick-born zoonotic pathogen but it may be also transmitted to human through direct contact with tissues and body fluids of infected individuals or animals [7,8]. The incubation period for CCHF disease is usually few days, and then infected patients may experience non-specific symptoms like fever, myalgia, diarrhea, nausea and vomiting. And some of the infected patients can proceed into the hemorrhagic phase of CCHF disease where they suffer from bleeding from different parts of the body [9]. Poor clinical prognosis of CCHF disease can be predicted when patients have high viral load, elevated level of inflammatory mediators, thrombocytopenia and absence of early antibodies response [6]. The diagnosis of CCHF disease is usually affirmed by employing a polymerase chain reaction (PCR) test that can measure the viral load in viremic phase of the disease [10]. Also, serological tests can be used to detect anti-CCHFV immunoglobulins. But these serological tests shouldn't be employed in the acute phase of CCHF disease because of the possible delay of antibodies response in infected individuals [6]. CCHFV is considered a public health threat due to the high case fatality ratio of CCHF disease, geographic widespread distribution of the vector and absence of either specific antiviral drug or vaccine. In this regard, the only antiviral drug recommended by the World Health Organization (WHO) for CCHF disease management is ribavirin [1]. However, the clinical effectiveness of ribavirin in management of CCHF disease seems to be inconclusive [11]. It is worth to mention that a previous invitro study had proposed that chloroquine or chlorpromazine may have a synergistic effect against CCHFV when combined with ribavirin. Also, this invitro study found that both chloroquine and chlorpromazine may inhibit CCHFV infectivity by targeting viral entry to studied cell lines [12]. The RNA genome of CCHFV consists of three major segments and these are: the large segment which encodes for RNA-dependent RNA-polymerase, the medium segment that encodes for glycoprotein while the small segment encodes for nucleoprotein [13]. The RNA genome of CCHFV doesn’t exist alone but instead it is encapsulated by the nucleoprotein to generate a ribonucleoprotein (RNP) complex. Then, this RNP complex associates with RNA-dependent RNA-polymerase to produce an active template for RNA synthesis of the virus [14]. Therefore, the nucleoprotein of CCHFV is believed to be essential for viral replication and thus represents a potential target to design novel antiviral capable of impairing the binding between nucleoprotein and viral RNA. In this trend, a recent structure-based virtual screening study of PubChem library was able to identify several potential hits against nucleoprotein of CCHFV [15]. Also, a previous molecular docking and dynamics simulation study had suggested that the FDA approved antibiotics doxycycline and minocycline may have the capacity to inhibit CCHFV nucleoprotein [16]. Additionally, multiple immuno-informatics tools were employed by another in-silico study to identify potential epitopes of CCHFV nucleoprotein and ovarian tumor domain for potential use in vaccine designagainst this virus [17]. In this computational study, we have screened a library of FDA approved drugs against the nucleoprotein crystal of CCHFV strain Baghdad-12. The aim of this in-silico study is to repurpose FDA approved drugs against CCHFV through inhibition of nucleoprotein binding with viral RNA and thereby impeding CCHFV multiplication. The methodology of our computational study was guided by the findings of a previous study published in 2012. In that study, the authors were able to crystalize and characterize the nucleoprotein of CCHFV strain originally isolated from a fatal case of CCHF in Iraq in 1979 [14,18, 19]. After crystallization of CCHFV nucleoprotein, the authors of that study had used a minigenome system with mutation analysis to define RNA binding site on the surface of nucleoprotein crystal. According to that mutation analysis, five amino acids in CCHFV nucleoprotein were found to be essential for binding of viral RNA and these residues are: Lysine 90, Lysine132, Glutamine 300, Lysine 411 and Histidine 456.Also, electrostatic potential study had showed that these five key residues are located in a continuous positively charged region on the surface of nucleoprotein crystal [14], as can be seen in Figure 1(see PDF). Therefore, it is of interest to document the data for molecular docking analysis and dynamics simulation of nucleoprotein of Crimea-Congo hemorrhagic fever virus strain Baghdad-12 with FDA approved drugs for drug design and development.

## Materials and methods:

### Setting up a methodology plan for virtual screening:

An illustration for virtual screening study steps can be seen in Figure 2(see PDF). In summary, the screening study started with prediction of viral RNA binding site on the surface of nucleoprotein crystal and subsequently setting up of docking coordinates. Next, the library of FDA approved drugs was screened by molecular docking against binding site on the surface of nucleoprotein monomer. And from the top 30 hits of docking output, only those drugs that are reported to be relatively safe by clinical references were subjected to molecular dynamics (MD) simulation for 20 nanoseconds. Then, only those drugs with close proximity to binding site during initial simulation were submitted for another MD simulation that extended for 50 nanoseconds. Finally, the final potential hits were filtered out based on evaluation of mean ligand movement throughout simulation period.

### Prediction of potential binding pockets:

We have used DoGSiteScorer online tool to detect any potential binding pocket within chain A of nucleoprotein crystal (PDB: 4AKL) for CCHFV. This online tool has the ability to rank the detected binding pockets based on their surface area, volume and druggability score [20]. Then, location of the best potential binding pocket within nucleoprotein crystal and position of key residues for viral RNA binding from a previous study [14] were used together to setup docking coordinates for virtual screening study.

### Structure-based virtual screening of FDA approved drugs:

For this virtual screening study, we have used a drug discovery platform known as Mcule.com [21]. This online platform implements different programs like AutoDock tools and AutoDock Vina to facilitate structure-based virtual screening process [22, 23]. The methodology applied for this structure-based screening step is identical to what we had used in our previously published studies [24-28]. Concisely, the library of FDA approved drugs was downloaded as SDF file from ZINC 15 website [29]. This library of 1,615 approved drugs was then uploaded into Mcule.com platform. Also, we have uploaded only chain A of CCHFV nucleoprotein crystal with PDB code 4AKL [14]. After that, the structure-based virtual screening of these approved drugs was carried out by Mcule.com against nucleoprotein monomer. We have applied default parameters for this screening study but the coordinates for AutoDock Vina were (X: -34, Y: 20, Z: 18) and the area of binding pocket was (22*22*22) Angstrom. Lastly, the docking hits were ordered according to their minimum energy of binding. For this screening study, we have selected only the best 30 hits with least energy of binding for more assessment of clinical indications and relative safety by Medscape.com online reference [30]. And from these top 30 docking hits, only those drugs with relatively safety were then downloaded as ligand-target complex with least energy of binding pose. Both Discovery Studio Visualizer version 21.1.0 and PyMOL version 2.4.1 were used to visualize the downloaded drug-nucleoprotein docking complex [31,32].

### Molecular dynamics (MD) simulation study:

Molecular dynamics simulation was first applied for 20 nanoseconds duration to the best docking hits that have a relative clinical safety. Then, only those hits that were able to maintain close proximity to nucleoprotein binding pocket were then submitted to a second MD simulation that lasted for 50 nanoseconds. The aim of running this second MD simulation was to ensure that these hits will be able to keep a close proximity to nucleoprotein binding site throughout 50 nanoseconds period. The software YASARA Dynamics v20.12.24 was used to carry out MD simulation study [33]. For each hit submitted to MD simulation, a PDB file of drug-nucleoprotein docking complex was used with least energy of binding pose. The options and parameters used to execute this MD simulation by YASARA Dynamics were similar to what we had applied in our previously published articles [25-28]. In summary, A concentration of 0.9% of NaCl was applied during simulation and an addition of either sodium ions or chloride ions were added to the system to ensure neutralization of ligand-nucleoprotein complex. And to remove any possibility of clashes throughout MD simulation, both steepest descent and simulated annealing minimizations were utilized. Also, both optimization of hydrogen bonds and prediction of pKa value were applied in order to fine-tune amino acid residues protonation atphysiologic pH [34]. The following force fields were employed during MD simulation: AMBER14 for solute, TIP3P for water, AM1BCC and GAFF2 for ligand [35- 37]. Due to the employment of Particle Mesh Ewald algorithm, no cutoff limit was applied for electrostatic forces [38]. While a cutoff limit of 8 Angstrom was used for van der Waals forces [39]. Also at a temperature of 298K and a pressure of 1 atm, equations of motions were utilized as multiple timesteps of 1.25 femtoseconds and 2.5 femtoseconds for bonded and non-bonded interactions respectively [40]. Lastly, we have used GraphPad Prism version 8.0.2 to plot and evaluate Root Mean Square Deviation (RMSD) of ligand movement during simulation period.Then, YASARA Dynamics software was used to compute Molecular Mechanics Poisson-Boltzmann Surface Area (MM-PBSA) binding energy for each drug-nucleoprotein complex. YASARA Dynamics can calculate MM-PBSA binding energy by utilizing AMBER14 force field, a built-in macro in YASARA Dynamics can fully automate calculation process. According to the guideline of YASARA Dynamics, the more positive MM-PBSA binding energy indicates better interactions between drug and target [41,42]. The YASARA Dynamics depends on the following equation to calculate MM-PBSA binding energy:

Binding Energy= EpotRecept + EsolvRecept + EpotLigand + EsolvLigand - EpotComplex - EsolvComplex

## Results and Discussion:

At first, we have submitted chain A of CCHFV nucleoprotein to DoGSiteScorer grid-based online tool in order to predict potential binding pockets within target crystal [20]. This grid-based tool was able to detect a number of binding pockets within nucleoprotein monomer, and these predicted pockets were ordered according to their surface area, volume and druggability score. Here, we reported only the best three binding pockets predicted on the surface of nucleoprotein monomer as seen in Table 1(see PDF). Also, the location of these three binding pockets within CCHFV nucleoprotein chain A is illustrated in Figure 3(see PDF). As noted in Table 1 and Figure 3(see PDF), the first pocket has the largest size, surface area and almost the highest druggability score. Interesting, the five key residues for RNA binding on surface of nucleoprotein are located in the first potential pocket [14]. Therefore, pocket number 1 was used to setup docking coordinates for the screening of FDA approved drugs against CCHFV nucleoprotein. After virtual screening of 1,615 FDA approved drugs against CCHFV nucleoprotein monomer, the docking hits were ranked based on their minimum energy of binding. As seen in Table 2(see PDF), only the best 30 hits of docking study were presented. Again, these top 30 hits were ordered in Table 2(see PDF) based on their least energy of binding. Then, Medscape.com online reference was used to report clinical indications for these hits in Table 2(see PDF). According to Medscape.com, many of these hits are psychoactive or antineoplastic agents with serious adverse effects and thus seem to be ineligible for further investigations in this virtual study. We also precluded Deferasirox and Tolvaptan from any additional computational evaluation due to their effects on essential metals and body electrolytes. Additionally, both Lumacaftor and Ivacaftor were eliminated from any further virtual assessment in this study due to their possible adverse effect on hepatic enzymes [30]. As such, only twelve potential hits with relative clinical safety were then subjected to molecular dynamics (MD) simulation for 20 nanoseconds. These twelve potential hits are Nebivolol, Naldemedine, Midazolam, Dolutegravir, Butenafine, Permethrin, Desloratadine, Azelastine, Dasabuvir, Loratadine, Darifenacin and Ketotifen. Of these hits, Dolutegravir and Dasabuvir are approved antiviral agents. As can be seen in Table 2(see PDF), the fourth hit Nebivolol had been reported to possess a moderate ability to block SARS-CoV-2 infection according to invitro study [43]. Interestingly, four antihistamine agents were presented in Table 2(see PDF) and these are Desloratadine, Azelastine, Loratadine and Ketotifen. And according to several invitro studies, these reported antihistamine drugs may be able to interfere with SARS-CoV-2 capacity to invade susceptible cells [44-46]. Also, the opioid receptor antagonist Naldemedine appeared in Table 2(see PDF) as the sixth best hit. Naldemedine may be able to block SARS-CoV-2 capacity to infect host cells as mentioned by a recent computational study [47]. Finally, the antimuscarinic drug darifenacin can be seen by the end of Table 2(see PDF), this potential hit together with Nebivolol are believed to be effective inhibitors against several conserved targets of SARS-CoV-2 like nucleoprotein and main protease according to a computational study [48]. Next, analysis of MD simulation results for these twelve drugs throughout 20 nanoseconds had showed that only Nebivolol and Loratadine can maintain a close proximity to nucleoprotein binding pocket as reported in Table 2(see PDF). It is well-known that low mean ligand movement RMSD throughout simulation duration indicates a close proximity to target binding site and this refers to stronger interaction between ligand and target. And by superposing the ligand-target complex on its reference structure throughout 20 nanoseconds simulation period, we had found that the mean ligand movement RMSD for Nebivolol and Loratadine was 3.86 and 3.24 Angstrom respectively. And through visualization of electrostatic potential for the docking complex between nucleoprotein monomer and Nebivolol in Figure 4 (A)(see PDF) or Loratadine in Figure 4 (B)(see PDF), we can clearly notice that both drugs were able to bind to RNA binding pocket on the surface of nucleoprotein as represented by the blue colored region. As seen in Figure 4(see PDF), both Nebivolol and Loratadine were involved in multiple interactions with amino acids located in RNA binding region of CCHFV nucleoprotein. Of interest, is the ability of Loratadine to form a carbon-hydrogen bond with Lysine 411 residue in nucleoprotein monomer? As mentioned previously, Lysine 411 is considered one of the key residues that are essential for viral RNA binding to CCHFV nucleoprotein [14]. Finally, both Nebivolol and Loratadine were challenged for their ability to interact with CCHFV nucleoprotein through a molecular dynamics simulation that lasted for 50 nanoseconds. As can be seen in Figure 5(see PDF), both drugs were able to keep a relatively close proximity to RNA binding pocket in nucleoprotein crystal throughout simulation period with a reported mean ligand movement RMSD of 5.043 and 4.287 Angstrom for Nebivolol and Loratadine respectively. Also, the calculated average MM-PBSA binding energy for Nebivolol against nucleoprotein monomer was -43.89 Kcal/ mol while the binding energy reported for Loratadine was -28.23 Kcal/ mol. As mention by YASARA guideline, the more positive MM-PBSA binding energy refers to better interaction and binding between ligand and target [42]. As such, Loratadine has a more positive average MM-PBSA binding energy throughout simulation which indicates a more favorable interaction with nucleoprotein monomer.

## Conclusion:

We report that the beta receptor blocker Nebivolol and the antihistamine Loratadine may have the potential to bind to nucleoprotein crystal of Crimea-Congo hemorrhagic fever virus strain Baghdad-12 and thereby impeding viral replication.
